# How a Hat May Affect 3-Month-Olds' Recognition of a Face: An Eye-Tracking Study

**DOI:** 10.1371/journal.pone.0082839

**Published:** 2013-12-11

**Authors:** Hermann Bulf, Eloisa Valenza, Chiara Turati

**Affiliations:** 1 Department of Psychology, University of Milano-Bicocca, Milano, Italy; 2 Department of Developmental and Socialization Psychology, University of Padova, Padova, Italy; 3 Interdepartmental Center for Cognitive Science, University of Padova, Padova, Italy; Monash University, Australia

## Abstract

Recent studies have shown that infants’ face recognition rests on a robust face representation that is resilient to a variety of facial transformations such as rotations in depth, motion, occlusion or deprivation of inner/outer features. Here, we investigated whether 3-month-old infants’ ability to represent the invariant aspects of a face is affected by the presence of an external add-on element, i.e. a hat. Using a visual habituation task, three experiments were carried out in which face recognition was investigated by manipulating the presence/absence of a hat during face encoding (i.e. habituation phase) and face recognition (i.e. test phase). An eye-tracker system was used to record the time infants spent looking at face-relevant information compared to the hat. The results showed that infants’ face recognition was not affected by the presence of the external element when the type of the hat did not vary between the habituation and test phases, and when both the novel and the familiar face wore the same hat during the test phase (Experiment 1). Infants’ ability to recognize the invariant aspects of a face was preserved also when the hat was absent in the habituation phase and the same hat was shown only during the test phase (Experiment 2). Conversely, when the novel face identity competed with a novel hat, the hat triggered the infants’ attention, interfering with the recognition process and preventing the infants’ preference for the novel face during the test phase (Experiment 3). Findings from the current study shed light on how faces and objects are processed when they are simultaneously presented in the same visual scene, contributing to an understanding of how infants respond to the multiple and composite information available in their surrounding environment.

## Introduction

Recognizing, identifying, and responding appropriately to different faces is a crucial cognitive achievement of our species. This sophisticated competence refers to the ability to discriminate between different exemplars of the face category, to recognize a face as familiar. It rests on recognition memory competencies and differs from face detection, which refers to the capacity to perceptually discriminate between a face and a non-face visual object. 

 Evidence reveals that, already at birth, infants not only differentiate between faces and non-face visual objects [1-6], but also process information about individual faces [7-11]. Three-day-old neonates are capable of discriminating between their mother’s face and an unfamiliar female face [7-10]. Such recognition ability can also be generalized to unfamiliar faces. After being habituated with a photograph of a stranger’s face, newborns show a visual preference for a novel face even after a 2 min retention interval [11]. This evidence converges to suggest that from birth infants are able to acquire and retain some visual information embedded in a face that allows subsequent recognition. 

Recent studies have shown that infants’ face recognition is based on a robust face representation that is resilient to a variety of facial transformations. Three- to 4-month-old infants recognize the invariant aspects of a face over changes of viewpoint [12], and motion [13]. Even newborns are capable of face recognition despite rotations in depth [14], occlusion [15], and deprivation of inner/outer features [16]. A different kind of transformation that faces might undergo derives from external elements, such as the hats or glasses that faces are frequently dressed-up with. By varying the appearance of a face, such external elements, defined as paraphernalia, might have an impact on an infant’s overall facial representation. 

 Many developmental studies have investigated the effect of paraphernalia on the recognition of a face by a child [17-23]. In such studies, children are asked to recognize a previously seen face that has been modified by the addition or removal of an item such as a hat or glasses. Young children experience great difficulty with this task, but from about 10 years of age it does not cause much of a problem. Accordingly, when asked to choose between a target originally seen wearing a hat but now hatless and a distractor with a hat, young children often incorrectly identify the latter as the target face. This paraphernalia effect has been interpreted as suggesting that young children are adversely affected by the inclusion of paraphernalia in the recognition task to a greater extend than are older children [19,21]. The effect of paraphernalia is reduced when the familiar and novel faces are highly dissimilar [20], when paraphernalia do not serve a discriminative function [17], or when the angular size of the stimuli is increased [23].

While many studies have investigated the effect of paraphernalia on face recognition in children, to the best of our knowledge only one study has explored infants’ ability to recognize a face in the presence of irrelevant paraphernalia [22], and demonstrated that face recognition in 1-year-olds was modulated by pre-exposure to the irrelevant paraphernalia information prior to the recognition task. Individuating whether face recognition in young infants might be affected by the presence of the additional information provided by paraphernalia is highly relevant given that most of the previous studies used prevalently artificial experimental manipulations. To overcome this drawback it is crucial to explore infants’ ability to recognize a face in perceptual conditions that more closely resemble the circumstances that naturally occur in everyday visual contexts, when, for example, human faces appear with extra features such as glasses, a hat or earrings. 

The current study used a visual habituation task to investigate whether and how 3-month-old infants’ ability to encode (i.e, habituation phase) and to recognize (i.e, test phase) the invariant aspect of a face is modulated by the presence of an add-on hat. In Experiment 1, the type of hat did not vary between the habituation and the test phases, and both the familiar and the novel faces wore the same hat during the test phase. Experiment 2 was identical to Experiment 1 except that during the habituation phase the face did not wear a hat. In Experiment 3, a competition between the identity of the face and the type of hat was introduced, with the novel face wearing the hat presented during habituation and the familiar face wearing a new hat. 

 Moreover, using an eye tracker, we examined how long the infants attended to face-relevant information (i.e., the top and the bottom parts of the face) compared to the paraphernalia (i.e., the hat). The eye tracker recorded the infants’ eye movements within 3 areas of interest (AOI). In all of the experiments the three AOIs corresponded to the hat, and the top and the bottom halves of each face. This allowed us to determine whether a variation in the appearance of a face with an external element, during the encoding and/or the recognition processes, produced an effect on infants’ visual exploration of the face stimuli and, in turn, on infants’ face recognition. 

## Experiment 1

**Figure 1:**
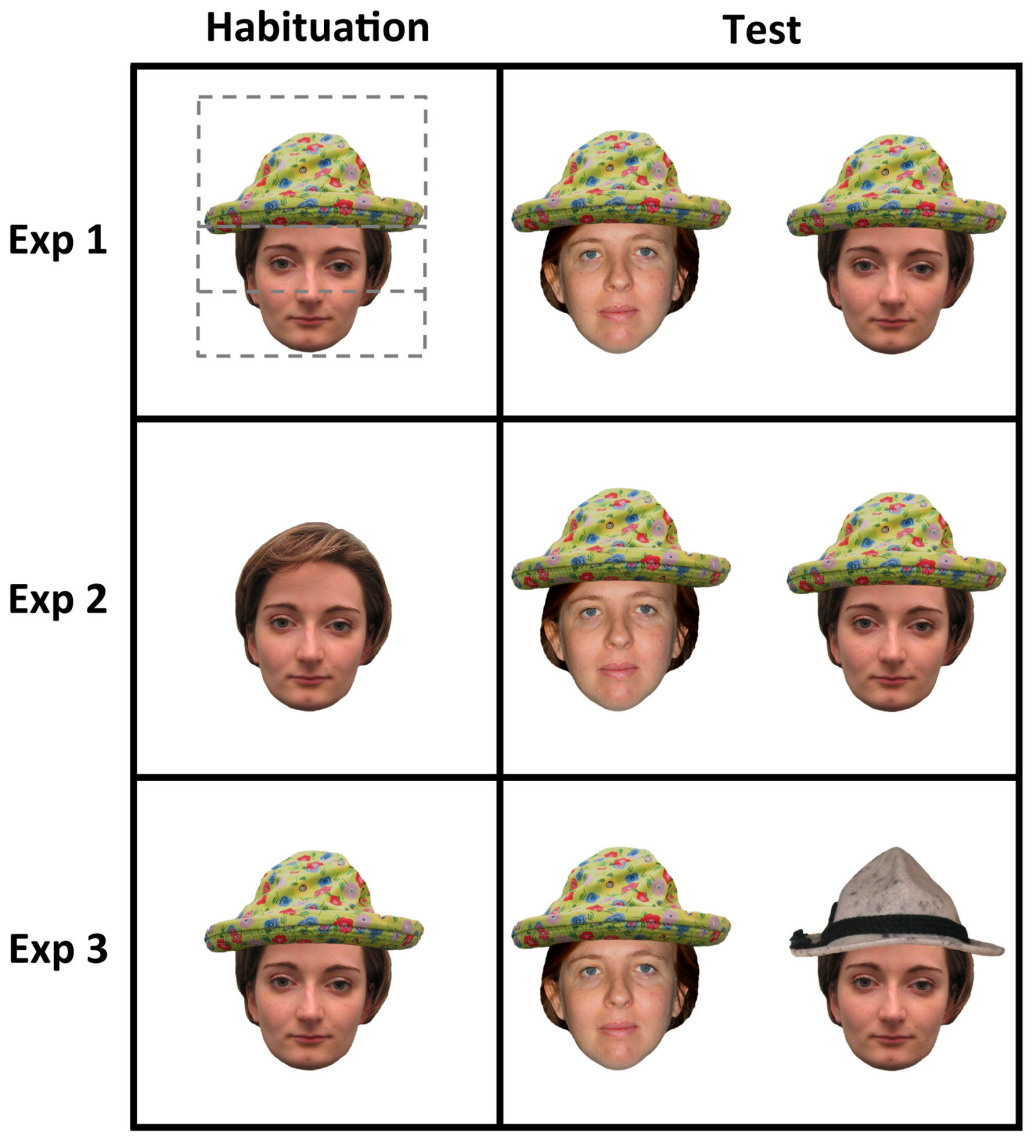
Examples of the stimuli used in the three experiments during the habituation and test phases. With dashed lines, the AOI relating to the hat and the top and bottom part of the faces. The subjects of the photograph have given written informed consent, as outlined in the PLOS consent form, to publication of their photograph.

 Experiment 1 tested whether the presence of external paraphernalia interfered with face recognition in 3-month-old infants. After being habituated to a face wearing a hat, the infants were presented with the familiar face paired with a novel face, both wearing the same hat presented during the habituation (Figure 1). In this way, while a change in the identity of the face was introduced, the hat was maintained constant between the habituation and test phases. A preference for the novel face would indicate that the infants were able to recognize the face presented during the habituation phase, overcoming the presence of the hat. Conversely, 3-month-old infants’ representation of the faces may be adversely affected by the presence of an external add-on element, which might interfere and prevent the infants’ face recognition processes.

### Method

#### Ethics Statement

The ethical committee of the Department of Developmental Psychology-University of Padova approved the present study.

#### Participants

The participants were 11 3-month-old healthy and full-term infants (7 females, mean age 99.7 days, range = 90-108). Four additional infants were tested but not included in the final sample due to a strong position preference (i.e., they looked in one direction more than 85% of the time during the test phase, n = 2) and fussiness (n = 2). The infants were tested only if awake and in an alert state, and after the parents had given their written informed consent. 

#### Stimuli

The faces were digitalized, high-quality colored images of 6 Caucasian female full-front faces, posing with a neutral expression and paired into three invariable pairs. All of the faces were unfamiliar to the participants. The face images measured about 14.5° in height and 11°-12° in width. The hats were digitalized, high-quality colored images of 3 different hats, measuring about 7.5° in height and 11-16° in width. The hats were superimposed on the faces using Adobe Photoshop CS3 (Figure 1).

#### Apparatus

The stimuli were presented with the software E-Prime 1.1 on a 19-inch monitor with a resolution of 1024 X 768 pixels. A remote, pan-tilt infrared eye-tracking camera (Model 504, Applied Science Laboratories [www.a-s-l.com], Bedford, MA) using bright-pupil technology was placed directly below the stimulus screen, and recorded the participants’ eye movements at a temporal resolution of 50 Hz. An experimenter guided the eye-tracking camera by means of a remote control, keeping the eye of the participant in focus. The image of the eye was viewed on a television monitor to simplify this procedure. Plain curtains were hung on both sides of the testing area to prevent interference from irrelevant stimuli. Behind the curtains were two computers: one generated the stimuli, and the other controlled the eye-tracking camera and collected the eye movement data. To coordinate the eye movement data with the respective stimulus displays, the stimulus-generating computer sent unique, time-stamped numerical codes via a parallel port to the data-collecting computer, indicating the onset and type of stimulus display. The digital data, indicating the fixation locations and changes in locations of the eye, were calculated in relation to the centroids of the pupils and the corneal reflections using the Applied Science Laboratories' algorithm.

#### Procedure

The experiment started with a calibration phase immediately followed by the habituation phase and the test phase. The eye tracker was calibrated by having the participant look at stimuli (animated cartoons with musical soundtracks) presented at three different locations on the stimulus monitor (center, top left, and bottom right). Calibration accuracy was checked and repeated if necessary. All subsequent eye data were calculated from these calibration values. The infants were not restrained in any way. The presence of dynamic cartoons with musical soundtracks during the calibration engaged the infants’ interest without any other restrictions. An infant control habituation paradigm was used. During the habituation trials, the infants were familiarized with a face wearing a hat presented in the center of the screen. Each habituation trial began with a central cartoon animated image to catch the infants’ attention. A soon as the infant fixated the central cartoon for 500 ms, the software automatically turned off the cartoon and activated the stimulus. When the infant looked away from the stimulus for more than 2 s, the image was automatically turned off and the cartoon animation reappeared on the screen to re-attract the infant’s attention. The habituation phase ended when the sum of the infant’s looking times on three consecutive trials was equal to or less than 50% of the total looking time from the infant’s first three trials [24]. In the test phase, each infant was given two 10 s paired presentations of the test stimuli. During each presentation, the infants were shown the image of the familiar face paired with a novel face, with both faces wearing the same hat presented in the habituation phase. The left-right position of the familiar and novel test faces was counterbalanced across the infants in the first test trial and reversed in the second test trial. 

#### Data analysis

The image of each face wearing the hat was divided into three AOIs, corresponding to the hat, and the top and the bottom halves of the face (Figure 1). The AOI corresponding to the hat measured about 9° in height and 17° in width, the AOI corresponding to the top half of the face measured about 4.5° in height and 17° in width and the AOI corresponding to the bottom half of the face measured about 4.5° in height and 17° in width. The total fixation times on the stimuli (calculated by summing all the fixations within the AOIs) and the percentages of the looking time within each AOI were computed for both the habituation phase and the test phase.

### Results

 All of the infants reached the habituation criterion. The mean total fixation time during the habituation phase was 53 s (SE = 6.42 s). The percentages of the looking time spent in each AOI are shown in Table 1. One-sample *t* tests were conducted to compare the percentages of the looking time in each of the three AOIs to the chance level, using Bonferroni adjusted alpha levels of 0.017 per test (0.05/3). Given that the AOI area corresponding to the hat was twice the AOI area corresponding to the two halves of the face, the chance level was calculated based on the percentage of the area each AOI occupied (see Table 1). The comparison was significantly different from the chance level for the top, *t*(10) = 4.21, *p* = 0.002, and the bottom part of the face, *t*(10) = 4.09, *p* = 0.002, and the only AOI significantly above chance being the one corresponding to the top part of the face (*M* = 57%). 

**Table 1:** Percentage of looking time (SE) and chance levels for each AOI in the habituation and the test phases of the three experiments. The numbers in bold indicate the percentages of looking time significantly different from chance.

Exp	AOI	Habituation phase		Test phase		
		% looking time	Chance level	% looking time novel face	% looking time familiar face	Chance level
1	Hat	33 (8.7)	50%	22 (7.5)	14 (3.2)	25%
	Face top	57 (7.6)	25%	28 (4.6)	19 (3.9)	12.5%
	Face bottom	9 (3.9)	25%	12 (6.8)	5 (2.0)	12.5%
2	Hat	21 (5.4)	50%	22 (4.6)	14 (2.4)	25%
	Face top	71 (5.3)	25%	32 (3.6)	24 (3.3)	12.5%
	Face bottom	8 (2.6)	25%	5 (2.0)	3 (1.0)	12.5%
3	Hat	39 (8.3)	50%	27 (4.8)	30 (3.5)	25%
	Face top	52 (7.0)	25%	21 (4.0)	14 (3.1)	12.5%
	Face bottom	9 (4.2)	25%	5 (2.9)	3 (2.0)	12.5%

**Figure 2:**
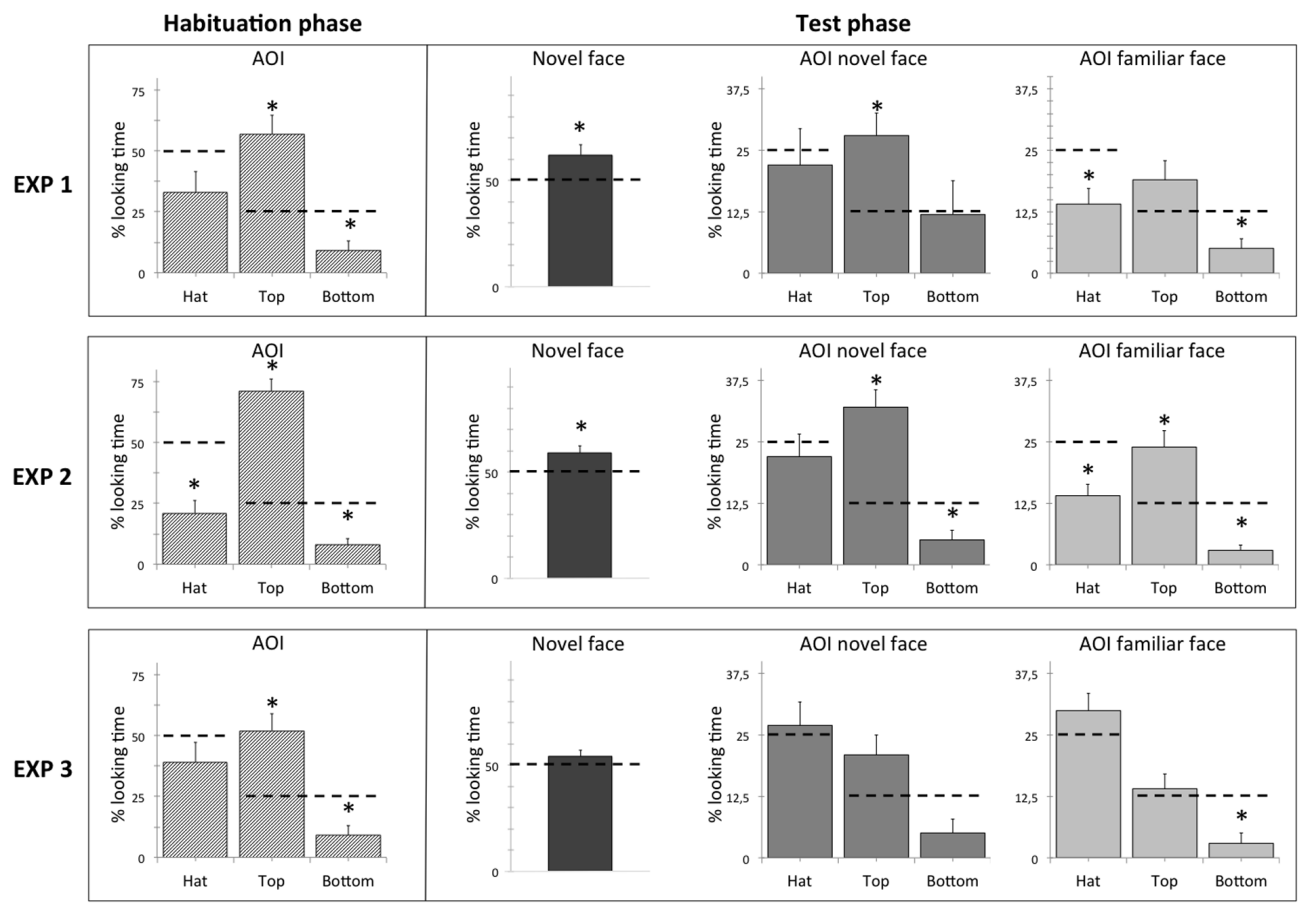
The percentages of looking time in the three experiments during the habituation and the test phase. Dashed lines indicate chance levels. The asterisks indicate the percentages of looking time that significantly differed from chance levels.

 We subsequently analyzed the total fixation times during the test phase, in order to test whether the infants were able to recognize the face to which they were habituated, irrespective of the presence of the hat. The infants looked longer at the novel face image (M = 6.67 s, SE = 0.597 s) than at the familiar one (M = 4.01 s, SE = 0.562 s), paired-samples *t*(10) = 2.59, *p* = 0.027. A novelty preference score (percentage) was also calculated. Each infant’s looking time at the novel face image during the two test presentations was divided by the total fixation time for both test stimuli, and subsequently converted into a percentage score. The novelty preference score (M = 62%, SE = 4.9%) was significantly different to the chance level of 50%, one-sample *t*(10) = 2.52, *p* = 0.03. The percentages of the looking time spent in each AOI are shown in Table 1. One-sample *t* tests were run to compare the percentages of the looking time in each of the six AOIs to the chance level, using Bonferroni adjusted alpha levels of 0.008 per test (0.05/6). Only the percentages corresponding to the top part of the novel face (28%), the hat of the familiar face (14%), and the bottom part of the familiar face (5%) were significantly different from the chance level, one-sample *t*(10) = 3.45, *p* = 0.006, *t*(10) = 3.58, *p* = 0.005, and *t*(10) = 3.55, *p* = 0.005 respectively (Figure 2), and the only AOI significantly above chance being the one corresponding to the top part of the novel face.

### Discussion

 Experiment 1 was aimed at investigating whether 3-month-old infants were able to recognize a face in the presence of an add-on element, i.e. a hat. The infants recognized the familiar face presented during habituation, spending more time looking at the novel face. Our findings provide evidence that, at 3 months of age, the presence of a hat does not interfere with either the encoding or the recognition of a face, at least when the same hat was unvaryingly presented in both the habituation and test phases. This conclusion is supported by the fact that the percentage of the looking time spent in the AOIs containing the hat did not differ from the chance level, either in the habituation phase, or in the test phase, although the AOI area containing the hat was twice the size of the AOI area containing the top and bottom part of the face. Indeed, visual exploration by the infants was focused on the top part of the face during habituation, and on the top part of the novel face during the test phase. These were the only AOIs that were explored with a percentage of the looking time above the chance level. 

 This outcome suggests that 3-month-old infants’ face recognition remains a stable process even in the presence of external paraphernalia. However, one may claim that the observed infants’ ability to recognize the familiar face during the test phase relied on a simple pattern-matching process. Actually, the face wearing the hat shown during habituation exactly matched the familiar stimulus presented in the test phase. In order to rule out the possibility that recognition rested on the infants’ ability to match two identical patterns, in Experiment 2 the faces wore the hat in the test phase, but not in the habituation phase. Therefore, the infants had to recognize a target face originally seen hatless, but now wearing a hat.

## Experiment 2

 In Experiment 2, during the habituation phase the infants were presented with an image of a face that did not wear a hat. The hat was shown only in the test phase, when both the familiar and the novel faces wore the same hat (Figure 1). Thus, unlike Experiment 1, the recognition of the familiar face could not be based on a simple image-based representation. We reasoned that, compared with Experiment 1, infants should have more difficulty in recognizing the familiar face because the stimulus encoded during the habituation phase differed from both the stimuli presented in the test phase. Therefore, the inclusion of a hat in the test phase might affect the infants’ recognition process, reducing the visual preference for the novel face found in Experiment 1 to the chance level. 

### Method

#### Participants

The participants were 17 three-month-old healthy and full-term infants (8 females, mean age = 99.2 days, range = 89-106 days). Six additional infants were tested but were not included in the final sample due to strong position preference (i.e., they looked in one direction more than 85% of the time during the test phase, n = 3), and fussiness (n = 3). The infants were tested only if awake and in an alert state and after the parents had given their written informed consent.

#### Stimuli, apparatus and procedure

The stimulus, apparatus and procedure were the same as those described in Experiment 1, except that the face presented during habituation did not wear a hat. Although during the habituation phase the hat was absent, the total fixation time and the percentage of the looking time were computed for the AOI corresponding to the hat for both the habituation and the test phase, using the same areas and criteria employed in Experiment 1.

### Results

 All of the infants reached the habituation criterion. The mean total fixation time during habituation was 46.06 s (SE = 5.07 s). Note that, unlike Experiment 1, the hat was not present here. However, for the sake of clarity, the label ‘hat’ was maintained for the corresponding AOI. One-sample *t* tests were run to compare the percentages of the looking time in each of the three AOIs to the chance level, using Bonferroni adjusted alpha levels of 0.017 per test (0.05/3). The percentages of the looking time spent in each AOI are shown in Table 1. The comparison was significantly below the chance level for the hat 21%, *t*(16) = 5.4, *p* = 0.001, and for the bottom part of the face 8%, *t*(16) = 6.48, *p* < 0.001. The only AOI significantly above the chance level was the one corresponding to the top part of the face (71%), *t*(16) = 8.7, *p* < 0.001, meaning that infants spent most of their looking time fixating on this AOI during the habituation phase. 

 As in Experiment 1, subsequent analyses aimed to test whether the infants were able to recognize the face to which they were habituated, irrespective of the presence of the hat. In the test phase, the infants looked longer at the novel image (M = 7.7 s, SE = 0.87 s) than at the familiar one (M = 5 s, SE = 0,6 s), paired-samples *t*(16) = 2.64, *p* = 0.018. A novelty preference score (percentage) was also calculated. The obtained novelty preference scores of 59% (SE = 3,4%) was significantly different to the chance level of 50%, one-sample *t*(16) = 2.7, *p* = 0.016. The percentages of the looking time spent in each AOI are shown in Table 1. One-sample *t* tests were run to compare the percentages of the looking time in each of the six AOIs to the chance level, using Bonferroni adjusted alpha levels of 0.008 per test (0.05/6). The percentages of looking time at the top part were significantly above the chance level for both the novel (32%) *t*(16) = 5.44, *p* < 0.001 and the familiar face (24%) *t*(16) = 3.38, *p* = 0.004. The percentages of looking time were significantly below the chance level for the bottom part of the novel face (5%) *t*(16) = 3.69 *p* = 0.002, as well as for the hat (14%) *t*(16) = 4.34 *p* = 0.001 and the bottom part (3%) *t*(16) = 8.51, *p* < 0.001 of the familiar face (Figure 2). 

### Discussion

 Experiment 2 was designed to investigate whether infants were able to recognize a familiar face wearing a hat after being habituated to the same face presented without the hat. Both the familiar and novel faces shown during the test phase wore the same hat. The results demonstrate that the infants looked more at the novel face than at the familiar one, providing evidence that they were able to recognize the face shown during the habituation phase. As in Experiment 1, the hat did not affect the infants’ ability to process face information. The percentages of the looking time spent in the AOIs containing the hat during the test phase did not differ from the chance level, although the AOI area containing the hat was twice the size of the AOI area containing the top and bottom part of the face. Moreover, the top part of the novel face was explored with a percentage of looking time above the chance level. Unlike Experiment 1, the infants’ visual exploration was also focused on the top part of the familiar face, with a percentage of looking time above the chance level. This difference between Experiment 1 and Experiment 2 is probably due to the fact that the presence of a modification between the habituation and the familiar test stimulus forced the infants to compare the familiar and novel face more strictly than in Experiment 1, in which the hat was unvaryingly encountered both in the habituation phase and in the test phase. Overall, these data provide evidence that 3-month-old infants are able to recognize a familiar face from a novel one even if a distracting add-on element is introduced during the recognition process. In addition, our outcome demonstrates that infants’ recognition ability cannot be reduced to a simple pattern matching process, because there was a mismatch in the presence or absence of the paraphernalia between the encoding phase (i.e., habituation) and the recognition phase (i.e., test). Nevertheless, the possibility remains that infants’ recognition performance is challenged when the paraphernalia serves a discriminative function. Thus, in Experiment 3, we manipulated both the novelty of the face and the novelty of the hat, with the novel face wearing the hat presented during habituation, and the familiar face wearing a new hat. In this way, unlike Experiments 1 and 2, the information concerning the hats was misleading and not consistent with the face information. This, in turn, might render the recognition task much more challenging due to the interaction between the salience of the information conveyed by faces and objects.

## Experiment 3

In Experiment 3 infants were habituated to a face that wore a hat. In the test phase, a novel face wearing the familiar hat was shown, together with the familiar face wearing a new hat (Figure 1). Since we manipulated both the novelty of the hat and the novelty of the face, we hypothesized that the paraphernalia would trigger the infants’ attention more than in the previous two experiments, interfering with the infants’ exploration of the face-relevant information. If this were the case, the infants might spread out their fixations to compare the novel and the familiar hat as well as the face specific information. In turn, this might challenge the infants’ face recognition competences. 

### Method

#### Participants

The participants were 15 three-month-old healthy and full-term infants (6 females, mean age = 100.8, range = 91-109 days). Five additional infants were tested but were not included in the final sample due to strong position preference (i.e., they looked in one direction more than 85% of the time during the test phase, n = 2), and fussiness (n = 3). The infants were tested only if awake and in an alert state and after the parents had given their written informed consent.

#### Stimuli, apparatus and procedure

The stimulus, apparatus and procedure were the same as those described in Experiment 1, except that during the test phase the novel face wore the familiar hat presented during habituation, and the familiar face wore a new hat (Figure 1). 

### Results

 All of the infants reached the habituation criterion. The mean total fixation time during the habituation phase was 54.35 s (SE = 6.5 s). The percentages of the looking time spent in each AOI are shown in Table 1. One-sample *t* tests were run to compare the percentages of the looking time in each of the three AOIs to the chance level, using Bonferroni adjusted alpha levels of 0.017 per test (0.05/3). The percentages of looking time were significantly below the chance level for the AOIs corresponding to the bottom part of the face (9 %), *t*(14) = 3.7, *p* = 0.002. The only AOI significantly above chance was the one corresponding to the top part of the face (52%), *t*(14) = 3.8, *p* = 0.002. To compare the percentages of looking time across the experiments we ran an ANOVA with AOI type (hat, top, bottom) as the within factor and Experiment (Experiment 1, 2, 3) as the between factor. The analysis revealed a main effect of AOI type, F(2,80) = 35.16, *p* < 0.001, since the infants looked longer at the AOIs containing the top part of the faces (61%) than at the AOIs containing the hats (30%), *t*(42) = 3.83, *p* < 0.001 and the bottom parts of the faces (8.8 %), *t*(42) = 11.62, p < 0.001. Also, the looking times were greater toward the AOIs containing the hats than toward the AOIs comprising the bottom parts of the faces, *t*(42) = 3.92, *p* < 0.001.

 Data analyses on the infants’ looking behavior in the test phase aimed to test whether the infants were able to recognize the face to which they were habituated, irrespective of the presence of the familiar hat. The Infants did not look significantly longer at the novel face (M = 8.2 s, SE = 0.5 s) than at the familiar face (M = 7 s, SE = 0.45 s), paired-samples *t*(14) = 1.48, *p* = 0.16. The novelty preference score (54%) (SE = 2.9), calculated as in previous experiments was not significantly different to the chance level of 50%, one-sample *t*(14) = 1.39, *p* = 0.185. The percentages of the looking time spent in each AOI are shown in Table 1. One-sample *t* tests were run to compare the percentage of looking time in each of the six AOIs to the chance level, using Bonferroni adjusted alpha levels of 0.008 per test (0.05/6). These showed that only the percentage of looking time at the bottom part of the familiar face was different from chance (3 %), *t*(14) = 6.07, *p* < 0.001, being below the chance level (Figure 2). 

 This finding provides evidence that the infants did not show a preference for the novel face when the novelty of the face competed with the novelty of the hat. To better understand the effect of an add-on element on infants’ face recognition a direct comparison between the results obtained in each of the three experimental situations employed in the current study is crucial. Specifically, it would be interesting to understand whether the presence of a novel hat in the test phase of Experiment 3 affected the infants’ fixation time differently to Experiments 1 and 2, where the hat was present, but did not serve any discriminative function. With this aim, we ran an ANOVA with Novelty (novel face, familiar face) and AOI type (hat, top, bottom) as within factors and Experiment (Experiment 1, 2, 3) as the between factor. The analysis revealed a main effect of Novelty, F(1,40) = 16.21, *p* < 0.001, and a main effect of AOI type, F(2,80) = 20.76, *p* < 0.001. Moreover, there was a significant interaction between AOI type and Experiment, F(4,80) = 2.66, *p* = 0.039. Post-hoc comparisons revealed a significant difference in the percentage of looking time toward the AOIs containing the hats between Experiment 3 (57%) and Experiment 2 (36%), *t*(30) = 2.24, *p* = 0.032, and a marginal significant difference between the AOIs containing the hats between Experiment 3 (57%) and Experiment 1 (36%), *t*(24) = 1.96, *p* = 0.06. Moreover, there was a significant difference between the looking time at the AOIs containing the top parts of the faces in Experiment 2 (56%) and Experiment 3 (35%), *t*(30) = 2.77, *p* = 0.009.

### Discussion

 The results of Experiment 3 show that, while during habituation the infants’ visual exploration was focused on the AOI containing the top part of the face, during the test phase infants explored the AOIs containing the novel and familiar hats as well as the top and bottom parts of the novel and familiar faces with a percentage of looking time that was not above the chance level. This finding provides evidence that, when the novelty of the face competed with the novelty of the hat, the infants spread out their fixations to compare the novel and familiar hat as well as the face specific information. As a consequence, the percentage of looking time toward the novel face did not differ from the chance level. 

 This lack of novelty effect can be interpreted as the infants’ inability to recognize the identity of the familiar face presented during habituation. Indeed, unlike Experiments 1 and 2, in the present experiment the information concerning the hats was misleading and not consistent with the face information. The competition between the novel face and the novel hat might have disrupted the infants’ face recognition capacity. If this were the case, the infants’ failure to recognize the familiar face cannot be explained as the infants’ inability to efficiently encode the information related to facial characteristics during the habituation phase, since the distribution of the infants’ looking time between the hat and the face during habituation was similar to that found in Experiment 1, in which the infants were able to recognize the familiar face, even in the presence of the add-on item. 

 Nevertheless, the lack of a preference effect in Experiment 3 does not necessarily imply an infants’ inability to recognize the face shown during the habituation phase. For example, it is possible that the infants recognized the familiar face, but simultaneously noticed the novel hat on the familiar face. Indeed, as revealed by the ANOVA run to compare the percentage of looking time across experiments, the distribution of looking time differed across the three experiments. In Experiment 3 the infants spent more time looking at the AOI containing the hats and, in turn, spent less time looking at the top part of the faces. In other words, the infants were drawn to both the novel face wearing the familiar hat and the novel hat worn by the familiar face, but their interest in both canceled each other out. When the visual scene comprises multiple novel objects, infants may need more time to process them. Thus, a possibility is that, in the case of a competition between the identity of the face and the type of hat, infants might need more than two 10 s paired presentations of the test stimuli to manifest a visual preference for the novel face. 

 Regardless of the interpretation for the lack of preference for the novel face during the test phase, the results from the present experiment provide evidence that the hats triggered the infants’ attention more than in the previous two experiments, leading to an interference effect between the information conveyed by the novel and familiar hats, and by the novel and familiar faces on the deployment of visual attention by the infants.

## General Discussion and Conclusions

The ability to recognize faces is a remarkable human feat. Here, we investigated how 3-month-old infants’ ability to recognize an unfamiliar face is vulnerable to an extraneous visual stimulus, i.e. a hat. Using an eye-tracker system in a visual habituation task, three experiments examined the impact of the add-on hat on face recognition. The use of the eye-tracker allowed us to record how long the infants attended to face-relevant information (i.e., the top and the bottom parts of the face) compared to the paraphernalia (i.e., the hat). The presence/absence of the hat during the habituation phase (face encoding) and the test phase (face recognition) was also manipulated. 

The three-month-old infants’ face recognition abilities were preserved when the novel and the familiar face wore the same hat during the test phase, both when the hat was unvaryingly shown during the habituation phase and the test phase (Experiment 1) and when the hat was shown only during the test phase (Experiment 2). These results suggest that infants’ face encoding and recognition processes are resilient to the presence of an add-on distracting element, as revealed by the fact that in both experiments the percentage of looking time spent in the areas of interest containing the hat was not above the chance level either during the habituation phase or the test phase. These findings are in line with the available evidence that 1-year-old infants are capable of recognizing a face wearing paraphernalia when the visual angular size of the stimuli is increased [22], our stimuli being comparable in dimension to those presented in the large visual angle condition by Lundy [22]. Moreover, our results appear consistent with studies demonstrating that the effect of paraphernalia on face recognition by children is reduced when the paraphernalia do not serve a discriminative function [17]. 

 When objects are presented as elements that remain constant throughout the recognition task, rendering the information conveyed by the objects of no use for discrimination purposes, identity recognition by infants is not influenced by the mere presence of the surrounding objects. On the contrary, when the novel face identity competed with a novel hat, with the novel face wearing the hat presented during habituation and the familiar face wearing a new hat (Experiment 3), the infants spread out their fixations to compare the novel and familiar hat as well as the face specific information. Thus, infants’ discrimination abilities seem to be determined by the interaction between the salience of the information conveyed by faces and objects. 

 Overall, our results suggest that infants are capable of recognizing the invariant aspects of a face thus creating a robust face representation even in the presence of an add-on hat. However, when the distracting item serves a discriminative function - i.e. the novelty of the hat competes with the novelty of the face - the hat triggers an infants’ visual attention, interfering with the amount of looking time spent on face information. The findings from the current study shed light on how faces and objects are processed when they are simultaneously presented in the same visual scene, contributing to an understanding of how an infant responds to the multiple and composite information available in its surrounding environment, which often differs significantly from the artificial stimuli employed to examine the emergence and development of cognitive processes in the first months of life [25].
